# Transfusional malaria in the neonatal period in Lagos, South-West Nigeria

**DOI:** 10.1371/journal.pone.0195319

**Published:** 2018-04-03

**Authors:** Franca Ogechi Iheonu, Iretiola Bamikeolu Fajolu, Chinyere Veronica Ezeaka, Wellington Aghoghovwia Oyibo

**Affiliations:** 1 Department of Paediatrics, Lagos University Teaching Hospital, Idi-Araba, Lagos, Nigeria; 2 Department of Paediatrics, College of Medicine, University of Lagos, Lagos, Nigeria; 3 Department of Medical Microbiology and Parasitology, College of Medicine, University of Lagos, Lagos, Nigeria; Food and Drug Administration, UNITED STATES

## Abstract

**Background and objectives:**

Sick neonates in malaria endemic areas are frequently transfused with donor blood unscreened for malaria parasite. Consequently, they are at risk of transfusional malaria which can lead to increased neonatal mortality. The study aimed to determine the burden of transfusional malaria in neonates to help in policy formulation on prevention of transfusional malaria.

**Materials and methods:**

One hundred and sixty four neonates admitted into the neonatal unit of a tertiary hospital over a 10 month period who were scheduled for blood transfusion were screened for malaria parasites pre-transfusion, at three and 14 days post transfusion using Giemsa stained thick and thin films. Donor blood was screened for malaria parasites at the point of transfusion. Neonates who developed malaria parasitaemia post transfusion were followed up for signs of malaria.

**Results:**

All recruited neonates tested negative to malaria parasite pre- transfusion. One hundred and twenty (73.2%) were term neonates with 94(57.3%) aged 1-7days. Four (2.4%) neonates developed malaria parasitaemia three days post transfusion and all four developed fever that resolved on treatment for malaria. Three (1.8%) of 164 donor blood samples had malaria parasitaemia and all three (100%) neonates who were transfused with the infected donor blood developed malaria parasitaemia post transfusion. However, one neonate who developed malaria parasitaemia post transfusion was transfused with non-infected donor blood.

**Conclusions:**

The prevalence of transfusional malaria in this study is low (2.4%). However, 100% of neonates who received malaria infected donor blood developed transfusional malaria. We therefore recommend routine screening of donor pre-transfusion, testing of neonates who develop fever post transfusion and treatment of those who test positive to malaria.

## Introduction

Blood transfusion is a lifesaving procedure that involves introduction of blood and blood products into the circulatory system of an individual. It is a common procedure in hospitalized children under the age of five years in malaria endemic countries because of their vulnerability to severe anaemia.[1] Other groups of children often transfused are those with hematological conditions, cancers and sick neonates. Sick neonates are intensively transfused due to common underlying conditions such as severe unconjugated hyper bilirubinaemia, neonatal sepsis, bleeding disorder, prematurity and anaemia from other causes. In Nigeria, 30% of neonates admitted into the neonatal intensive care units (NICU) receive one or more transfusions prior to discharge[2,3].

Even though blood transfusion can be lifesaving, it is associated with complications such as transmission of infections, immunological reactions and volume overload. Infective agents that can be transmitted to recipients of blood transfusion includes; viruses, bacteria, spirochetes and protozoa. Transfusional malaria (TM) is the most common protozoal transfusion transmissible infection in malaria endemic countries[4] and it is defined as the presence of malaria parasite in the peripheral blood of an individual whose peripheral blood was previously negative for malaria parasite prior to transfusion.[5] Transfusional malaria is different from malaria acquired through mosquito bite; in the later, sporozoites injected into the blood stream by the mosquito firstly undergo development in the liver (pre-erythrocytic schizogony) and then in the red blood cell (erythrocytic schizogony) with an incubation period of 7-35days. In contrast, in TM, blood stage parasites (trophozoites, gametocytes) introduced into the blood stream undergo erythrocytic schizogony immediately. The liver stage is by-passed therefore, incubation period of TM is short, relapse does not occur and malaria parasites can multiply rapidly after a blood transfusion leading to severe infection.

Sick neonates who undergo blood transfusion in sub-Saharan Africa especially in Nigeria are at risk of TM. This is because several studies have shown that the prevalence rate of malaria parasitaemia in donor blood across the SSA countries and in Nigeria is high with a range of 0.7%-55%[6] and 4.7%-74.1%[7–11] in SSA and Nigeria respectively. Also, routine screening of donor blood for malaria parasites prior to transfusion is currently not being practiced. Studies have shown that recipients of infected donor blood developed malaria parasitaemia post transfusion.[12–14] This growing evidence of high burden of TM has not been backed up with policy formulation on prevention of TM in most countries with a high burden of malaria including Nigeria. Consequently, clinicians rely on individualized practices such as administration of anti-malaria medication to recipients of blood transfusion which may not be evidenced based and cost effective.[7,15] This practice can also worsen the emerging resistance to artemisinin based combination therapy (ACT). Therefore, there is need for more data on TM to guide on policy formulation to standardize practice.

Furthermore, the aforementioned studies were conducted in a mixed population of adult and children and so the findings may be different in the neonatal population especially with the recent report of increasing prevalence of neonatal malaria.[16,17] Also, malaria in the neonatal period has been reported to have similar symptomatology with neonatal sepsis which is a common cause of hospital admission in neonates.[18] Therefore, TM in neonates may be missed or undiagnosed leading to fatal outcome. It is therefore necessary to determine the burden of transfusional malaria in the neonatal population as this will fill the existing gaps in knowledge and provide scientific information for policy formulation/implementation.

The aim of this study was to determine the incidence and risk factors of transfusional malaria in neonates in South-West Nigeria.

## Materials and methods

### Study design

This cross sectional descriptive study was carried out at the neonatal intensive care unit of a tertiary hospital in South-West Nigeria between February and November 2015.

### Study location

Lagos University Teaching Hospital has two NICU; in-born and out-born units for admitting neonates delivered within the hospital and those referred from other facilities respectively. The hospital has a blood bank that runs 24-hours services providing screened and cross-matched blood products when required. Blood is obtained from voluntary donors, families and friends who are routinely screened using questions and temperature check to exclude high risk donors who may transmit infections and potential donors with a recent history of fever; temperature greater than 37.5°C; symptoms of ill health, current or recent use of malaria medications in addition to those who have risky behavior are excluded from being donors. Donors are also routinely screened for HIV, hepatitis B, hepatitis C and syphilis prior to donation. Malaria parasite is not routinely screened for in the donor blood prior to transfusion.

### Enrolment

Admitted neonates who were scheduled for whole blood or packed red cell transfusion and who satisfied the following criteria were consecutively recruited into the study: mothers/care givers gave written informed consent, were negative when tested for malaria parasites prior to transfusion, were not on malaria medication and not on breast milk from mothers on malaria medication within three days pre or post-transfusion. Neonates who received more than a blood component and had multiple transfusions where excluded. A pretested questionnaire (S1 Appendix) was used to obtain information on demographics, feeding option and use of malaria medication from mothers/caregivers. Details of blood transfusion such as indication for transfusion, type of transfusion, type of blood component, duration of storage of donor blood and blood group were obtained from the case notes of the neonates. Blood transfusion types were; (1) top-up transfusion with 10-15mls/kg of packed red cells or 20mls/kg of whole blood, (2) single exchange transfusion with 85mls/kg of whole blood and (3) double volume exchange transfusion with 170mls/kg of whole blood. Donor blood was either fresh (donated on the same day of request and lasting less than 24hours from the time of donation to the time of transfusion) or stored under refrigeration at +2 to +6°C (for 24hours or more from the time of donation to transfusion). Duration of storage was mostly less than five days in line with the guideline for neonatal transfusion which recommends fresh blood. Packed red cells were obtained on request from whole blood by separation method. They were neither washed, irradiated nor leukocytes reduced. Blood sample was obtained from the blood bag at the point of transfusion and from heel pricks of neonates prior to transfusion and 72 hours post-transfusion for malaria microscopy. Blood sample was also collected from neonates who developed parasitaemia three days post transfusion on day 14 post transfusion for microscopy.

### Ethical approval

Approval for the study was obtained from the Lagos University Teaching Hospital Health Research and Ethics Committee (HREC). A written informed consent was obtained from the mothers/care givers of the neonates and information obtained was handled confidentially. Feedback on the test result was communicated to the mothers/care givers. The neonates were followed up post transfusion and the result of those with malaria parasitaemia was promptly made available to the managing physicians for appropriate treatment.

### Laboratory procedure

Thick and thin smears were prepared from blood samples obtained from the blood bag and from neonates pre transfusion, three days and 14 days post transfusion. The smears were prepared and subsequently stained with freshly prepared Giemsa solution according to standard procedure.[19] The stained slides were each read twice independently by two malaria microscopists from the WHO certified malaria research laboratory at the College of Medicine, Lagos for concordance. Any discordance in result was resolved by re-examination of the slides by a third microscopist who acted as a tie breaker. (shown on S2 Appendix)

### Follow up and treatment

Transfused neonates were followed up post transfusion for signs and symptoms attributable to malaria such as fever, vomiting, diarrhea, jaundice and refusal of feed. Axillary temperature of neonates was measured prior to transfusion and for 14 days (8hourly and any time the parent/care giver felt the neonate’s body was warm) post transfusion. Those who developed fever or symptoms were tested for malaria parasitaemia and those who tested positive were promptly treated and a repeat malaria microscopy carried out 14 days post-transfusion to determine parasite clearance.

### Data analysis

The data obtained was inputted and analyzed using the Statistical Package for Social Sciences (SPSS) version 20.0(Armonk, NY: IBM Corp). Frequency distribution tables and bar chart was generated for categorical data. Test of hypothesis for categorical data was done using the Chi-square and Fishers exact test. The Kolmogorov-Smirnov test was used to test for normality of continuous variables. Continuous variables were expressed as means and standard deviations when normally distributed or median and inter-quartile range when skewed. The Student’s t test was used to test for statistical difference between normally distributed continuous data. Spearman’s rank order correlation was used to determine the relationship between parasite density in neonates and parasite density in transfused blood. Binary logistic regression was used to determine the odd ratios and statistical significance of some predictors of TM. Level of significance was set at p value of <0.05.

## Results

One hundred and sixty four neonates concluded the study schedule; one hundred and thirty-three (81.1%) and thirty one (18.9%) were recruited from the out-born and in-born units of the hospital respectively. One hundred and four (63.4%) of the study participants were males with a male to female ratio of 1.7:1. The overall mean age was 9.1 ± 6.5 days, with 57.3% of the study population aged 1–7 days and 42.7% aged 8-28days. One hundred and twenty (73.2%) neonates were delivered at term and the mean gestational age was 37.0 ± 3.2weeks. Of the forty-four neonates delivered preterm, 21 (47.7%) were late preterm. The mean birth weight was 2656.9 ± 794.3 grams with 66.5% of the participants having normal birth weight. (Table 1)

**Table 1:** Socio-demographic characteristics of the study participants.

Variables	Frequency n = 164	Percentage (%)
**Sex**		
Male	104	63.4
Female	60	36.6
**Age (days)**		
1–7	94	57.3
8–28	70	42.7
**Maturity**		
Preterm (<37 weeks)	44	26.8
Term (37–41 weeks)	120	73.2
**Gestational age (weeks)**		
<28 (early preterm)	5	3.0
28- <32 (very preterm)	7	4.3
32 -<34 (moderately preterm)	11	6.7
34–36 (late preterm)	21	12.8
37–41 (Term)	120	73.2
**Birth weight (grams)**		
<1000 (extreme low birth weight)	5	3.0
1000 - <1500 (very low birth weight)	11	6.7
1500 - <2500 (low birth weight)	39	23.8
2500–4000 (normal birth weight)	109	66.5

### Indications for transfusion in the study participants

A total of one hundred and three (62.8%) of the participants were transfused on account of severe hyperbilirubinaemia; forty-five of these participants had both severe hyperbilirubinaemia and anaemia. Of the sixty-one participants who were transfused on account of anaemia from various causes, majority (47.5%) of them had sepsis as the cause of anaemia. (Table 2)

**Table 2:** Indications for blood transfusion in the study participants.

Indications for transfusion	Frequency N = 164	Percentage %
Severe hyperbilirubinaemia (only)	58	35.4
Severe hyperbilirubinaemia and anaemia	45	27.4
Anaemia from prematurity	9	5.5
Anaemia from sepsis	29	17.7
Anaemia from other causes (Cephal hematoma, post-circumcision bleed etc)	23	14.0

### Blood products and transfusion types in the study participants

One hundred and forty-six (89.0%) of the study population were transfused with whole blood while eighteen (11.0%) were transfused with packed cells. One hundred and thirty (79.3%) received stored blood while thirty-four (20.7%) received fresh blood. The median age of stored blood was 2.0 days with a range of 0 to 19 days. Most (62.7%) of the participants had double volume exchange transfusion. (Table 3)

**Table 3:** Blood products and transfusion types in the study participants.

Variables	Frequency n = 164	Percentage (%)
**Blood component**		
Whole blood	146	89.0
Packed cells	18	11.0
**Blood storage**		
Stored blood	130	79.3
Fresh blood	34	20.7
**Type of transfusion**		
Double volume EBT	103	62.8
Top-up transfusion	54	32.9
Single volume EBT	7	4.3

### Incidence of malaria parasitaemia in transfused donor blood

Three (1.8%) out of one hundred and sixty-four transfused donor blood samples used in the study were positive for malaria parasites. Trophozoites of *P*. *falciparum* were identified in all three transfused donor blood samples. The median parasite density was 525 parasites/μl of blood (range 30–6029).

### Incidence of malaria parasitaemia in participants three days post transfusion

It was observed that four (2.4%) out of one hundred and sixty-four participants developed malaria parasitaemia three days post transfusion and all had trophozoites of *P*. *falciparum*detected in their peripheral blood with median parasite density of 908 parasites/μl of blood (range 71–4079 parasites/μl of blood). All four implicated donor samples in post transfusion malaria were whole blood; three of which were fresh and one stored for five days. Two of the fresh donor samples (each 15mls and 20mls) were used for top-up transfusion in preterm neonates whiles the remaining two; one fresh (420mls) and one stored (500mls) were used for double volume EBT. One of the implicated donor sample (20mls) which was fresh and used for top-up transfusion was negative for malaria parasites.

### The association between malaria parasitaemia in neonates post transfusion with malaria parasitaemia in transfused donor blood

All three participants who received donor blood positive for malaria parasites developed malaria parasitaemia three days post transfusion while one (0.6%) of the remaining 161 participants who were transfused with malaria negative donor blood developed post transfusional malaria. The association between receiving malaria positive blood and development of post transfusional malaria in the neonates was found to be statistically significant (*p*<0.001). (Table 4)

**Table 4:** Association between malaria parasitemia in neonates post transfusion with malaria parasitaemia in transfused donor blood.

MP in donor blood	MP in neonates blood post transfusion		Total
	MP Negative neonates	MP Positive neonates	
MP Negative transfused blood	160	1	161
MP Positive transfused blood	0	3	3
Total	160	4	164

*P*< 0.001 by Fishers exact.

MP = Malaria parasites.

### Correlation of malaria parasite density in neonates post transfusion with parasite density in transfused donor blood

There was a positive and strong correlation between malaria parasite densities in neonates (recipients) three days post transfusion with malaria parasite density in transfused donor blood. (r = 0.862, p<0.001)

### Post transfusion malaria parasitaemia in participants in relation to factors such as demographics, blood products and transfusion types

A higher proportion of those who developed malaria parasitaemia post transfusion were very preterm (50%) and aged 1–7 days (75%). However, a statistically significant relationship was observed across gestational age (*p*<0.001) and birth weight (*p* = 0.022). (Table 5)

**Table 5:** Post transfusion malaria parasitaemia in the study participants in relation to demographic factors.

Factors		Malaria microscopy results post-transfusion			
	MP Negative n = 160 n (%)	MP Positive n = 4 n (%)	Total n = 164 n (%)		*P*value
**Gestational age(weeks)**					
Extreme preterm	5(3.1)	0(0)	5(3.0)		
Very preterm	5(3.1)	2(50)	7(4.3)		
Moderately preterm	11(6.9)	0(0)	11(6.7)		
Late preterm	20(12.5)	1(25)	21(12.8)		
Term	119(74.4)	1(25)	120(73.2)		<0.001*
**Birth weight(g)**					
ELBW	4(2.5)	1(25)	5(3.0)		
VLBW	10(6.3)	1(25)	11(6.7)		
LBW	38(23.7)	1(25)	39(23.8)		
Normal birth weight	108(67.5)	1(25)	109(66.5)		0.022*
**Sex**					
Male	102(63.8)	2(50)	104(63.4)		
Female	58(36.2)	2(50)	60(36.6)		0.624
**Age (days)**					
1–7	91(56.9)	3(75)	94(57.3)		
8–28	69(43.1)	1(25)	70(42.7)		0.637

*p-*values from Fishers exact test.

* = statistically significant.

MP = malaria parasite, LBW = low birth weight, VLBW = very low birth weight, ELBW = extreme low birth weight.

A higher proportion of participants who developed post transfusion malaria were transfused fresh blood (75%) and this relationship was statistically significant (*p* = 0.028). All the participants with post transfusion malaria were of blood group O and received whole blood but there was no statistically significant relationship between blood components received (*p* = 1.00), blood group (*p* = 0.319), type of transfusion (*p* = 0.724) and the development of malaria parasitaemia post transfusion. (Table 6)

**Table 6:** Post transfusion malaria parasitaemia in the study participants in relation to blood product and transfusion types.

Factors		Malaria microscopy result post transfusion				
	MP Negative n = 160 n (%)		MP Positive n = 4 n (%)	Total n = 164 n (%)		*P*value
**Blood storage**						
Stored	129(80.6)		1(25)	130(79.3)		
Fresh	31(19.4)		3(75)	34(20.7)		0.028*
**Blood component**						
Whole blood	142(88.8)		4(100)	146(89)		
Packed cells	18(11.2)		0(0)	18(11)		1.00
**Types of transfusion**						
Double volume EBT	101(63.1)		2(50)	103(62.8)		
Top-up transfusion	52(32.5)		2(50)	54(32.9)		
Single volume EBT	7(4.4)		0(0)	7(4.3)		0.724
**Blood group**						
O+	89(55.6)		3(75)	92(56.1)		
O-	5(3.1)		1(25)	6(3.7)		
A+	25(15.6)		0(0)	25(15.3)		
A-	5(3.1)		0(0)	5(3.0)		
B+	34(21.3)		0(0)	34(20.7)		
AB+	1(0.6)		0(0)	1(0.6)		
AB-	1(0.6)		0(0)	1(0.6)		0.319

*P* values from Fishers exact test.

* = statistically significant.

EBT = exchange blood transfusion.

### Predisposing factors to transfusional malaria

Binary logistic regression analysis using the demographic and hematological factors as the independent variable and the presence or absence of post transfusion malaria in the study participants as the dependent variable is shows that LBW and transfusion with fresh blood were significant predictors of post transfusion malaria in the study participants. Participants with ELBW were 27 times more likely to develop TM compared with 11 times in the VLBW and three times in the LBW neonates. Participants who were transfused fresh blood were 12 times more likely to develop TM when compared to those transfused stored blood. (Table 7).

**Table 7:** Binary logistic regression model of the predictors of post transfusion malaria among the study participants.

Variables	B	S.E.	Wald	*p* value	Odds ratio	95% confidence interval	
						Upper limit	Lower limit
**Birth weight(g)**							
ELBW	3.296	1.503	4.808	0.028*	27.00	1.419	513.796
VLBW	2.380	1.452	2.684	0.101	10.80	0.627	186.058
LBW	1.045	1.427	0.536	0.464	2.84	0.173	46.567
Normal birth wt.	Ref	-	-	-	-	-	-
**Maturity**							
Preterm	2.164	1.169	3.428	0.064	8.707	0.881	86.056
Term	Ref	-	-	-	-	-	-
**Age (days)**							
1–7	0.822	1.166	0.497	0.481	2.275	0.232	22.344
8–28	Ref	-	-	-	-	-	-
**Sex**							
Female	0.565	1.013	0.310	0.577	1.759	0.241	12.818
Male	Ref	-	-	-	-	-	-
**Blood storage**							
Fresh blood	2.524	1.172	4.640	0.031*	12.48	1.256	124.128
Stored blood	Ref	-	-	-	-	-	-

*Ref* = reference group.

* = statistically significant.

wt. = weight.

## Discussion

This study has shown that neonates transfused with unscreened donor blood are at risk of TM and that 100% of neonates transfused with malaria infected donor blood will develop TM. Although 100% of neonates transfused with malaria infected blood developed TM, a low incidence rate of 2.4% was found in the study and this can be explained by the low prevalence rate (1.8%) of malaria parasitaemia in the donor blood. A similar incidence rate of 2% was reported by Owusi-Ofori*et al*[13] in a mixed population of children aged 1–15 years and adults but a slightly higher incidence rate of 3.5% was reported by Ali *et al*[12] in a similar population of children and adults. This observed difference may be due to the relatively lower prevalence rate (1.8%) of malaria parasitaemia in the donor blood recorded in the present study compared to 6.5% reported by Ali *et al*[12] and also due to the different population studied.

Prevalence rate of 1.8% for malaria parasitaemia in donor blood observed in the current study is lower than the previous reports in Nigeria where a range of 4.1% in the South-Western[7] to 74.1% in the South-Eastern[9] part of the country had been reported but compares with reports of similar studies in Ethiopia and Nepal where prevalence rates of 1% and 1.3% respectively were reported.[20,21] The disparity in findings may be due to regional differences; climatic, environmental, socio-economic, culture, belief in Nigeria, difference in study designs and effective malaria control. In the index study, donor blood samples for malaria microscopy was collected from the blood bag at the point of transfusion unlike in the studies with higher prevalence rates[9,22] where donor blood samples were collected directly from the donors at the point of bleeding at presentation. Parasites in stored blood have been reported to lose their viability over time. This was pointed out by Chattopadhyay *et al*[23] who found out that parasitaemia was much lower in samples collected from blood bag stored over a period compared to when the same blood was tested at the point of donation. This may explain why the incidence of TM was significantly higher in neonates who were transfused freshly donated blood (p = 0.03) compared to stored blood. Therefore, it is necessary to determine the viability of malaria parasites in transfused donor blood stored over a range of period in our environment.

The decline in the prevalence of malaria parasitaemia in transfused donor blood recorded in the study may be a reflection of effective malaria control with the recent scale up in vector control through the use of insecticide treated mosquito nets (ITNs) and indoor residual spraying (IRS), community awareness programs, better diagnostics and use of artemisinin-based-combination therapy (ACT). The WHO reported a significant improvement in the use of ITNs, IRS, diagnostic testing for malaria with corresponding increase in the use of ACTs in Africa.[24] The report of the 2015 National Malaria Indicator Survey (NMIS) also showed increased household access and use of long lasting-insecticidal net (LLIN), diagnosis and treatment of malaria using RDT and ACT respectively.[25] The NMIS observed a rapid decline in malaria prevalence in children 6–59 months in South-Western Nigeria from 60.5% by RDTs and 50.3% by microscopy in 2010 to 1.9% by RDT and 0% by microscopy in 2015.[25] Therefore, the prevalence of 1.8% recorded in the current study may truly represent the dividend of effective malaria control. In practice, sustenance and consolidation of these gains in malaria control will further eliminate or reduce the prevalence of malaria parasitaemia in transfused donor blood and invariably reduce the risk of TM immensely.

A significant association was observed between malaria parasitaemia in transfused donor blood and parasitaemia in the neonates post transfusion. Similarly, the parasite density in the transfused donor blood correlated positively and strongly with parasite density in the neonates post transfusion. These findings support transmissibility of malaria parasites from asymptomatic blood donors to recipients of blood. All three participants who received malaria infected donor blood developed malaria parasitaemia post transfusion representing a 100% risk to the recipients. On the contrary, Ali *et al*[12] and Owusi-Ofori*et al*[13] reported that 14% of the participants in their study which were mainly adults and children developed malaria parasitaemia post transfusion. The higher risk observed in the current study may be due to the different population studied and the diagnostic tool used. Sick neonates may be more vulnerable to TM when compared to children and adults. Furthermore, microscopy which was used as the diagnostic tool in the current study is limited in specificity unlike PCR which can confirm that transfused parasite is responsible for malaria infection in the recipients as was demonstrated in the study by Owusi-Ofori*et al*.[13] Therefore, 100% risk recorded in the present study may be overestimated since it was assumed that identified parasites in the donor and the recipient’s blood were identical.

One participant (0.6%) who was transfused with freshly donated non-infected donor blood developed parasitaemia post transfusion. The likely reasons for this finding may be that malaria parasite infection was acquired through the bite of an infected mosquito and parasites were in the liver stage of development (pre-erythrocytic schizogony) during the initial microscopy pre-transfusion. The age of the participant (20 days) further supports acquired malaria as the mode of transmission excluding congenital malaria which is expected to occur in the first week of life. It is however possible that parasite in donor blood might have been missed by microscopy which has been reported to have low sensitivity at low parasite density. The non-use of polymerase chain reaction (PCR) as the diagnostic tool in the present study is a major limitation as it use may have increased the diagnostic yield.

The proportion of the participants who developed malaria parasitaemia post transfusion was significantly influenced by gestational age, birth weight and duration of storage of blood. On further analysis using logistic regression, it was observed that low birth weight (LBW) and fresh blood transfusion were predictors of TM. The higher risk of developing TM with decreasing birth weight is of public health concern considering the rapid increase in this population resulting from increased survival due to better neonatal intensive care. These neonates are particularly at greater risk because they are born earlier before maternal transfer of immunoglobulin. Therefore, they may be prone to severe TM. Participants who received fresh blood were observed to be 12 times more likely to develop TM when compared to those who were transfused stored blood. This finding portends a serious health implication for neonatal care where small volume transfusion with fresh blood is often practiced. Routine screening of freshly donated blood prior to transfusion is further emphasized.

It was observed in the study that even though the subjects who developed TM had normal temperatures pre-transfusion, all (100%) of the participants developed a fever post transfusion with a range of 37.7°C- 39.1°C recorded between days two and five post transfusion. Ali *et al*[12] reported a fever in 31.1% of their study population who were adults and children. This difference may be due to the different population studied. Neonates may not have developed defense mechanism to mount response to malaria parasites unlike in older children and adults who may have acquired protective immunity.

### Conclusions and recommendations

The study has shown100% concordance rate of malaria infection in neonates transfused with malaria infected donor blood. Also, neonates with low birth weight and transfused with fresh donor blood are more likely to develop TM. This high burden of TM in neonates necessitates policy formulation. The authors recommend routine screening of freshly donated blood prior to transfusion to neonates and children under 5 years and follow up of all recipients of blood transfusion. Also, future studies using PCR is recommended.

### Limitations

We were unable to use polymerase chain reaction which is a more sensitive and specific method for malaria parasite testing.

All blood donors over the study period could not be tested for malaria parasite at the same time point.

## Supporting information

S1 Appendix Study questionnaire. (DOCX)

S2 Appendix Malaria microscopy reading form. (DOC)
