# Supplementary material for: Transfusional malaria in the neonatal period in Lagos, South-West Nigeria
Source: PLoS One. 2018 Apr 3;13(4):e0195319. doi: 10.1371/journal.pone.0195319 (PMC5882143; doi:10.1371/journal.pone.0195319)
Supplement: S1 Appendix — (DOCX) [file pone.0195319.s001.docx]

**Transfusional Malaria in the Neonatal Period in Lagos South-West Nigeria**.

**Questionaire**

**(A) Infants data** (To be completed by the researcher/assistant).

1. Study ID_________ Hospital number ___________ Date of enrolment_________
2. Date of birth _____/____/_____ Sex: male[1] female[2]
3. Gestational age (weeks) ________ Maturity: term[1] preterm[2]
4. Birth weight (g) ____________ Location of care: in-born [1] out-born [2]
5. Diagnosis on admission ­­­­­­­­­­­­­­­­­­­­­­­­­__________________________ Pre-transfusion PCV(%)______________
6. Symptoms and signs prior to transfusion (please tick if present)

Fever[1] Vomiting[2] Diarrhea[3] Poor suck[4] Respiratory distress[5]

Jaundice[6] Excessive cry[7] Seizures[8] Hepatomegaly[9] Splenomegaly[10] Others(specify)_______________________________________

1. Axillary temperature just before transfusion in ^o^C ______ Current weight (g)­­­­­­­­­­­­­­­­­­­­­­­­­­­­­_________
2. Indications for transfusion (please tick as appropraite).

Sepsis[1] Hyperbilirubinemia[2] Bleeding disorder[3] Anaemia of prematurity[4] Anaemia[5] Others (please specify)[ ] __________________

1. Blood group (please tick as appropriate) O+[1] A+[2] B+[3] AB+[4] Others(specify)____
2. Type of blood product/transfusion. (please tick and indicate volume used in transfusion)

| Date/Time of transfusion | Whole blood  Used | Packed cells  Used | Top up transfusion | Single volume exchange | Double volume exchange |
| --- | --- | --- | --- | --- | --- |
|  |  |  |  |  |  |
|  |  |  |  |  |  |

1. Record of axillary temperature and new symptoms/signs post transfusion.

| Days | 1 | 2 | 3 | 4 | 5 | 6 | 7 | 8 | 9 | 10 | 11 | 12 | 13 | 14 |
| --- | --- | --- | --- | --- | --- | --- | --- | --- | --- | --- | --- | --- | --- | --- |
| T^o^C am  Pm |  |  |  |  |  |  |  |  |  |  |  |  |  |  |
| Symptoms  /signs |  |  |  |  |  |  |  |  |  |  |  |  |  |  |

**(B) Donors data**

1. Blood group O+[1] A+[2] B+[3] AB+[4] Others (specify) __________
2. Type of blood used stored blood[1] Fresh blood [ 2]
3. If stored blood is used, what is the duration of storage (days) ____

**(C) Demographics**

1. Mothers contact address__________ Maternal age (years)__________

2. Mothers/fathers telephone numbers:_____________

3. Tribe: [1] Ibo [2] Yoruba [3]Hausa [4]Others (please specify) ___________

4. Mother’soccupation:[1]Civil servant [2]Public servant [3]Self employed [4]Unemployed

5. Father’s occupation: [1]Civil servant [2]Public servant [3]Self employed [4]Unemployed

6. Mother’s highest level of education: Primary[1] Secondary[2] Tertiary[3]

7. Father’s highest level of education: Primary[1] Secondary[2] Tertiary[3]

8. Feeding option [1] breastfeeding [2] breast milk substitute [3] mixed

9. If breast feeding, has mother received any anti-malarial medication in the past 3 days?

10. Does mother have a fever Yes[ 1 ] No[ 2 ]
