# Supplementary material for: Transfusional malaria in the neonatal period in Lagos, South-West Nigeria
Source: PLoS One. 2018 Apr 3;13(4):e0195319. doi: 10.1371/journal.pone.0195319 (PMC5882143; doi:10.1371/journal.pone.0195319)
Supplement: S2 Appendix — (DOC) [file pone.0195319.s002.doc]

Malaria Microscopy Record

(Microscopist No.1/2, first/second read)*(Tick as applicable)*

**Study ID: _______________ Day of Visit:____(E.g D0, D3, D14) Date: ___/___/_______ (dd/mm/yyyy)**

White blood cell count (number of white cells per microlitre of blood): ___________________ (WBC/µL)

-------------------------------------------------------------------------------------------------------------------

### MICROSCOPY

**Count number of asexual parasites relative to 500 white blood cells; gametocytes against 1,000 wbc.**

**Count all species present and record separately gametocytes and asexual parasites.**

**Estimated wbc for parasite density determination: 8,000**

| **Species** | Pf | | Po | | Pm | | Pv | |
| --- | --- | --- | --- | --- | --- | --- | --- | --- |
|  | Asex | Gam | Asex | Gam | Asex | Gam. | Asex | Gam. |
| **Number of parasites counted** |  |  |  |  |  |  |  |  |
|  | | | | | | | | |
| Number of white cells counted |  |  | | | | | | |

Comments: ________________________________________________________________________

Initials: _________ Signature: _________________________ (of microscopist)

----------------------------------------------------------------------------------------------------------------------

### PARASITE DENSITY

**Calculate parasite density in number of parasites per microlitre of blood (para/µL).**

**Use number of asexual parasites only.**

**Estimated wbc for parasite density determination: 8,000**

**= para/µL**

**____________ x WBC/μL**

**White cells countd**

**Asex paras. counted**

SEXUAL (GAMETOCYTE PARASITAEMIA):_______________________ ***(parasites / µL of blood)***

## Parasite Density Calculations

Study ID:_______________ Day of Visit:____(E.g D0, D3, D14) Date: ___/___/_______ (dd/mm/yyyy)

------------------------------------------------------------------------------------------------------------

### PARASITE DENSITY CALCULATION

**Estimated wbc for parasite density determination: 8,000**

| Parasite Density (number of parasites / µL of blood) | Microscopist 1 | Microscopist 2 |
| --- | --- | --- |
| Calculate Mean Density: (Density 1 + Density 2) / 2 |  |  |
| **Calculate Discrepancy (%): (Diff/mean) x 100** |  | |
| **If discrepancy is ≤ 20%- Use mean parasite density.** | | |
| ***If discrepancy is >20% - Repeat Microscopy*** | | |
| Parasite Density (number of parasites / µL of blood) | Micro 1- 2^nd^ reading | Micro 2 -2^nd^ Reading |
| ***Of the 4 readings, choose (circle) the two closest (one each from the different microscopists)*** | | |

| Parasite Density (number of parasites / µL of blood) | Microscopist 3  (Third count) | Microscopist 4  (fourth count) |
| --- | --- | --- |
| Calculate Mean Density: (Density 1 + Density 2) / 2 |  |  |
| **Calculate Discrepancy (%): (Diff/mean) x 100** |  | |

**DETERMNED PARASITAEMIA – ASEXUAL:_________________ *(parasites / µL of blood)***

**DETERMINED PARASITAEMIA – SEXUAL: _________________ *(parasites / µL of blood)***
